# Professional development: a mixed methods study of Masters of Public Health alumni

**DOI:** 10.3389/fpubh.2024.1429474

**Published:** 2024-10-23

**Authors:** Orna Baron-Epel, Yana Douvdevany, Dana Ivancovsky-Wajcman, Paul Barach, Osnat Bashkin, Katarzyna Czabanowska, Keren Dopelt, Nadav Davidovitch, Szczepan Jakubowski, Fiona MacLeod, Maureen Malowany, Leah Okenwa-Emegwa, Maya Peled-Raz, Shira Zelber-Sagi

**Affiliations:** ^1^School of Public Health, Faculty of Social Welfare and Health Sciences, University of Haifa, Haifa, Israel; ^2^Population Health Science Department, College of Population Health Thomas Jefferson University, Philadelphia, PA, United States; ^3^Department of Surgery, Imperial College, London, United Kingdom; ^4^Research Institute for Health Law and Science, School of Medicine, Sigmund Freud University, Vienna, Austria; ^5^Department of Public Health, Ashkelon Academic College, Ashkelon, Israel; ^6^Department of International Health, Care and Public Health Research Institute (CAPHRI), FHML, Maastricht University, Maastricht, Netherlands; ^7^Department of Health Policy Management, Institute of Public Health, Jagiellonian University, Kraków, Poland; ^8^Department of Health Policy and Management, Guilford Glazer Faculty of Business and Management and Faculty of Health Sciences, Ben-Gurion University of the Negev, Beer Sheva, Israel; ^9^The Israeli Association of Public Health Physicians (IPAPH), Israeli Medical Association, Ramat-Gan, Israel; ^10^Department of Health Promotion and e-Health, Faculty of Health of Science, Jagiellonian University Medical College, Kraków, Poland; ^11^School of Public Health, University College Cork, Cork, Ireland; ^12^Hebrew University-Hadassah, Braun School of Public Health and Community Medicine, Jerusalem, Israel; ^13^The Swedish Red Cross University Stockholm, Huddinge, Sweden

**Keywords:** Masters of Public Health, alumni, mixed methods, professional development, competencies, higher education

## Abstract

**Introduction:**

We examined the perceptions of the Master of Public Health (MPH) degree graduates regarding their personal competencies, job performance and professional development using a mixed method, explanatory sequential design.

**Methods:**

A cross-sectional, self-administered questionnaire of the Haifa School of Public Health alumni who graduated between 2005 and 2022 was disseminated to 849 graduates between March and June 2022, from which 127 responded (response rate: 14.90%). This was followed by 24 in-depth interviews with alumni from the same sample (conducted between November 2022 and March 2023).

**Results:**

The sample included 74.8% of females with a mean age of 40.7 years, 35% of alumni agreed that the MPH degree helped them attain a promotion in their present position (in rank or salary), and 63.8% felt that the degree helped them improve their job performance and contribute to their current workplace. Most (80.3%) alumni reported not changing jobs after graduation. The interview themes revealed that the MPH contributed to their personal and professional lives, provided them with a holistic view of public health and health systems, and improved their in-depth scientific skills. The main reported barriers to professional development included missing core competencies, low salaries, and a lack of information regarding suitable jobs. Surprisingly, an MPH was not a requirement for some public health sector jobs. Alumni reported that the MPH degree contributed to improving many graduates’ careers and satisfaction levels and to build their leadership competencies in public health.

**Discussion:**

There seems to be a lack of coordination between the academic curriculum and the jobs available for alumni, hindering better alumni professional development. Regular discussions, information sharing, and curriculum refinements between MPH program leaders and health sector leaders might help address many of the concerns of MPH degree graduates.

## 1 Introduction

The central roles of the Public Health (PH) services in response to 21st century challenges have highlighted the need to maintain a highly effective PH workforce (PHW). The PHW across the world has had to react and adjust swiftly to existential challenges such as the pandemic, wars, and natural disasters, in addition to performing ongoing PH responsibilities (e.g., monitoring of infectious and chronic diseases, routine childhood vaccine programs) and controlling other emerging health threats (1). Yet, not enough is known in Israel and many other places about whether the outcomes and impacts of a Master of Public Health (MPH) degree training prepares graduates with the right competencies needed for the PHW and helps graduates secure fulfilling and effective jobs. The capacity-building in higher education project “Sharing European Educational Experience in Public Health for Israel” (SEEEPHI) was established to transfer knowledge and best practices from European Union Higher Education Institutions (HEIs) to countries and HEIs outside the EU. The project was funded through ERASMUS+, with The Association of Schools of Public Health in the European Region (ASPHER) as the coordinating organization (2).

A Master of Public Health (MPH) degree is expected to teach the essential competencies needed for graduates to perform their PHW tasks (3–5). The World Health Organization (WHO) Regional Office for Europe and ASPHER, define ten competency categories that focus on PH content and contexts, relations and interactions, performance, and achievement (6). The WHO-ASPHER Competency Framework for the Public Health Workforce in the European Region (CFPHW) provides standardization and consistent definitions for the competencies required by PH professionals. Most Schools of Public Health incorporate these into their study curricula. However, it is not clear how the MPH effectively prepares their graduates for lifelong satisfying jobs in the PHW and how the MPH sub-specializations address the public health needs of the 21^st^ century. This is important for low-and middle-income countries (LMICs) and high-income countries alike (7), and relates to the general debate on the relevance of higher education to society and specific professions and jobs (8).

Studies have shown that despite extensive efforts to define PH professional competencies in academic programs, PH graduates do not possess the real-world needed competencies as measured by the Essential Public Health Operations (9, 10). Measuring the outcomes and impacts of educational programs is fraught with methodological difficulties (11, 12). Zwanikken et al. (13) suggested that the impact of an MPH program can be conceptualized as “impact in the workplace” and “impact on society.” They surveyed graduates of MPH programs in six countries and reported that the MPH programs contributed to the graduates’ use of competencies especially in regards to the workplace and had less impact on society (7). They also found clear differences between MPH programs. The same authors in a follow-up qualitative study found considerable impacts on the workplace at the national level in two countries (13). Additional studies have looked at other types of related programs, such as Belkowitz et al. (14) who surveyed recent graduates of a four-year MD/MPH program and found many had fulfilling leadership roles in their careers, contributed to research and about one-third worked in public health during their residency.

This study aimed to identify and analyze MPH alumni perceptions of the impacts that their Master of Public Health degree had on graduates’ careers, the application of competencies acquired in the MPH degree and their workplace.

## 2 Methods

We conducted an exploratory prospective, mixed methods study as part of a multinational Erasmus Plus Capacity Building European Union grant in Higher Education entitled “Sharing European Educational Experience in Public Health for Israel (SEEEPHI): harmonization, employability, leadership, and outreach” (15).

### 2.1 Study design and population

This is a cross-sectional, mixed-methods explanatory sequential design study of the University of Haifa graduates. The Haifa School of Public Health is one of four graduate Schools of Public Health in Israel. In addition, there is one undergraduate program at Ashkelon College. The Haifa School of Public Health was founded in 2003 and has nine different sub-specializations, with approximately 120 new students each year in the MPH program and about seven new PhD students. The students are mainly health professionals, including nurses, physicians, nutritionists, and others. The students are mainly in their 30’s and are a few years after their undergraduate studies, therefore have a few years of experience as healthcare workers, a minority are not healthcare workers. In their undergraduate studies the vast majority of MPH students are directed at acquiring a healthcare profession, such as nursing, medicine, or nutrition, and they are not exposed to public health.

The healthcare professions the alumni acquired in their undergraduate studies are geared towards individual patient care and in contrast, the MPH studies provide a new outlook on health issues, looking at the community as a whole. The MPH studies at the University of Haifa are intended mainly for professionals in mid-career, working full-time in the healthcare system. The large majority of students study one day a week and the rest of the time are employed. In addition to the holistic public health perspective, alumni obtained skills and competencies they did not have before. The specific competencies obtained depended on the sub-specializations they chose. For example, in the ‘Nutrition, Health, and Behavior,’ sub-specialization, the curriculum contains courses related to nutritional therapy or cognitive behavioral therapy, and in the ‘Health Promotion’ sub-specialization, the students are planned to acquire competencies to develop and promote health interventions. The students decide to study MPH and which specialization to choose without any national (or other) directing or funding. There is no governmental or other support for MPH studies.

This study included a quantitative survey, conducted between March and June 2022, followed by qualitative interviews among a sub-sample, conducted between November 2022 and March 2023, of the MPH alumni who graduated from the Masters of Public Health degree program between the years 2005 and 2022.

### 2.2 Data collection

After obtaining ethics approval to contact alumni, the School of Public Health at the University of Haifa’s secretary sent an email in March 2022 to all graduates from 2005 to 2022. A reminder was sent in April 2022, and the survey closed in June 2022. Participation in the survey was voluntary, responses were anonymous, and participants had the option to withdraw at any stage. The questionnaire was disseminated to 849 graduates between March and June 2022, of which 127 responded (response rate: 14.90%).

At the end of the questionnaire, the alumni were invited to participate in an in-depth interview. Alumni agreeing to an in-depth interview completed a separate form providing their contact details and consent. A follow up letter was sent to those who consented and an explanation was provided of the goals of the qualitative study and an explanation about recording their consent at the beginning of the interview. In addition, direct contact was made with alumni who did not answer the questionnaire to better represent all sub-specialties and genders.

The qualitative data collection was performed until complete saturation was achieved. The in-depth interviews were conducted by a sole interviewer (YD), conducted remotely using Zoom video software, and lasted 30–40 min on average. The interviews were recorded and transcribed *verbatim*, with the recordings subsequently deleted to preserve the anonymity of participants. The video recording was deleted subsequent to the interview.

The core study group (OBE, YD, DIW, MPR, SZS) developed the survey and the interview protocol. The survey was pretested with four alumni and revised based on comments received. The questionnaires underwent face, content and consensual validity by the SEEEPHI participants. The survey was hosted on Google Forms and contained 16 open and multiple-choice questions. All questionnaires were anonymously collected and analyzed. The full questionnaires are available in Supplementary material S1.

The in-depth interview protocol was developed based on the survey’s findings. A semi-structured interview protocol including nine open-ended questions to elicit in-depth information regarding alumni’s feelings towards their MPH degree and its impact on their work, profession and satisfaction. The interviewer (YD), highly experienced in conducting interviews, prompted the interviewee with probing questions to further investigate their opinions and feelings.

### 2.3 Data analysis

The quantitative data was analyzed using Microsoft Excel software. Descriptive statistics included demographic data and frequencies of answers to the questions (i.e., the number of participants, their mean age, gender, and year of graduation).

The interviews were transcribed and analyzed by two researchers (YD and DIW) who performed a thematic analysis of the transcribed data. The analyses included both a deductive approach based on categories in the interview guide and an inductive approach that developed new categories and themes throughout the analysis process (16) A review of the interview scripts served as the basis for a preliminary category structure, followed by axial coding to identify relationships and patterns for theme development.

Furthermore, an expansion methodology was employed to address a central theme not covered in the questionnaire and that emerged prominently during the interviews, enriching the study’s findings with a broader perspective derived from the qualitative data analysis (17). Integration of the qualitative and quantitative data analyses was based on weaving narrative, joint display, and an integrated discussion by the research team (18). The qualitative data offered more detailed descriptions of the alumni’s experiences, while the quantitative data allowed for general and representative findings (19).

### 2.4 Ethics

The University of Haifa Ethics Committee granted ethical approval for the study, including the interview guide and participants’ information sheets, and waived the requirement of signed informed consent from the participants (approval #060/22).

## 3 Results

### 3.1 Demographics of respondents

The survey was sent to 849 graduating alumni between 2005 and 2022 from the graduate studies at the School of Public Health, University of Haifa. Of those, 127 alumni (14.9% response rate) answered the questionnaire. The mean age of the respondents was 40.73 ± 9.3 (range: 25–62), and 74.8% were women (*n* = 95) which is representative of the school’s student body having female majority, with an average percentage of women in the past 10 years of 78%. Most, 93.7% (*n* = 119), of the respondents graduated with a master’s degree, and 6.3% (*n* = 8) graduated with a PhD degree. Regarding the PH sub-specializations, 20.5% graduated from the Health Nutrition and Behavior program, 20.5% from the Health Systems Management program, and 59.0% from Public Health (i.e., epidemiology, health promotion, health system administration, etc.). This represents the distribution of students between the sub-specializations in the school. A total of 24 alumni (20 women and 4 men) agreed to be interviewed using in-depth interviews. One participant graduated with a PhD, and 23 graduated with an MPH; of those, 15 completed research projects and submitted a thesis to earn their degree. The remaining participants did not do a research thesis for their degree. Regarding the PH sub-specialization, 26.1% studied in the Health Nutrition and Behavior program, 26.1% in the Health Systems Management program, and 47.8% graduated from the other sub-specializations (Table 1).

**Table 1:** Demographics of study respondents.

Parameter	Quantitative survey (*n* = 127)	Qualitative interview (*n* = 24)
Age (years) mean ± SD	40.73 ± 9.32 (range: 25–62)	39.54 ± 6.61 (range: 27–61)
Gender (% female)	74.8% (*n* = 95)	83.3% (*n* = 20)
Graduation year (range)	2005–2022	2007–2022
The degree studied % (*n*)		
Master	93.7% (119)	96% (23)
PhD	6.3% (8)	4% (1)
MPH sub-specialization % (*n*)		
Health nutrition and behavior	20.5% (26)	26.1% (6)
Health systems management	20.5% (26)	26.1% (6)
Health promotion	22.8% (29)	26.1% (6)
Other programs	36.2% (46)	21.7% (5)
Present workplace % (*n*)^a^		
Hospital	40.2% (51)	37.5% (9)
Healthcare service (e.g., HMO)	22.8% (29)	54.1% (13)
Private clinics	15.0% (19)	8.3%, (2)
Other	31.5% (40)	20.8% (5)
The position in present workplace% (*n*)^a^		
Manager	26.8% (34)	25% (6)
Nurse	19.7% (25)	8.3% (2)
Physician	5.5% (7)	8.3% (2)
Nutritionist	18.9% (24)	25% (6)
Researcher (including in academia)	6.3% (8)	20.8% (5)
Teacher	3.1% (4)	0
Health promoter	1.6% (2)	16.6% (4)
Other	18.1% (23)	25% (6)
Undergraduate studies^a^		
Nursing	32.3% (41)	29.1% (7)
Nutrition	24.4% (31)	33.3% (8)
Medicine (M.D) or veterinary medicine	10.2% (13)	8.3% (2)
Biology, pharmacy or medical lab	8.7% (11)	16.7% (4)
Teaching	4.7% (6)	0
Sociology, anthropology or psychology	3.9% (5)	8.3% (2)
Engineering or computer science	3.1% (4)	0
Other	12.7% (16)	4.1% (1)

a Since some alums have multiple workplaces or multiple undergraduate studies, the percentage sum is higher than 100.

The data analysis resulted in four main themes and seven sub-themes that emerged from the interviews: (I) Impact of MPH studies on professional and personal development, (II) Impact of MPH studies on occupation (present job, promotion, increase in salary, and changing jobs), (III) Challenges and difficulties; and, (IV) Information Platform about Public Health Positions. Table 2 illustrates the integrated analysis of the quantitative and qualitative data, including the themes, sub-themes, and representative quotes related to the impacts of the MPH degrees.

**Table 2:** Summary and synthesis of the quantitative and qualitative data.

Theme	Sub-themes	Quantitative findings	Representative Quotes	Inferences
*Theme I* Impact of MPH studies on professional and personal development	1. The MPH studies provided a broader and more holistic view of public health	To what extent do the competencies (e.g., statistical analysis, scientific writing, critical science reading, etc.) you obtained during your studies help you perform your job? Very much 20.5% Moderately 31.5% Little 36.2% Not at all 11.8%	*“I gained more insight, a look at reality, at the long term regarding the health of the children I take care of. It’s not just physical, I look at them as a population that needs to take care of their health.”* Interview 18 “*Of the main courses, they are about health systems, how the health systems work… I’m already more proficient in understanding how they work, the bureaucracy, the ways and the processes, so it’s easier for me to understand.”* Interview 9	The interviewees reported on the contribution of their MPH program studies to their personal and professional lives. The main contribution to their present position was obtaining a more holistic and broader view of public health issues, scientific approach and specific competencies for public health.
	2. The MPH studies provided both professional and personal competencies		*“Yes, it’s another thing for my life, for my personality. It gave me more connections, it gave me the ability to deal with other things that I may be weak in… I do not think I was the same before the studies, it was important.*” Interview 3 *“First of all, it is the ability to study, the ability to sit down and learn and enter the unknown… This ability to study is very important, it is very critical. Even when you do a thesis, you learn a lot by yourself, you do not have someone to sit with you, you have general instructions and you continue with them. So there is something in the fact that you enter a new field and the self-learning in me jumped out*…” Interview 9	
*Theme II* Impact of MPH studies on graduate occupation	1. Application of competencies obtained during studies to present position	To what extent does the degree you studied help you contribute to your present workplace? Very much 33.1% Moderately 30.7% Little 25.2% Not at all 11.0%	*“Learning how to evaluate public health and health promotion in particular. I acquired this in the program and not in any other way… including the whole subject of identifying needs, getting to know the population and planning a health promotion program. I learned all this in the program.”* Interview 24 (Health promotion specialist) *“I consider myself a good therapist and I think it would not have been good enough without some of the courses I took in my degree. There were some courses that really opened my eyes and changed me and really helped me.”* (dietician working in hospital) Interview 21	The interviewees reported that scientific competencies contributed to their present position, as well as more orientation towards public health issues and systems. Specific competencies were especially important in health promotion and nutrition, health and behavior.
	2. Impact of studies on promotion at current job or increase in salary	To what extent did the degree you studied help you get a promotion (in rank or salary) in your current job? Very much 13.4% Moderately 22.0% Little 25.20% Not at all 39.40%	*“I made this change following my studies and then I moved to a more managerial and responsible level… Yes, I felt that without the master’s degree I would not have reached this position, because, for the expertise in a field, this position, this title, the master’s degree is required.”* Interview 9 *“Look, on the one hand, it’s interesting and challenging because you do develop the same workshops, and you really have an influence,* etc. *But on the other hand, another role is added to your role, which also takes a lot of time and a lot of energy, and in the end, you do not get any extra (payment).* *They gave me something else, it does not matter at all, not in terms of salary or anything, it’s like an additional position.”* Interview 15	Most alumni reported the MPH program/degree did not help them get a salary increase, nonetheless, they got a promotion or additional job. Approximately half of the interviewees reported that their MPH studies contributed to their present work position in various ways; however, they did not aid in securing another job.	
	3. Changing jobs	To what extent did the degree you studied help you find your present position? Very much 11.8% Moderately 13.5% Little 14.2% Not at all 60.6%	*“These courses help in the work itself, in working with people, in working in the clinic, but in terms of jobs, it does not open a new horizon.”* Interview 3 *“It sucks because I know I have the competencies and I know I’m professional and I know everything. But the problem is the terms of employment, how can that be?!”* Interview 3	Both quantitative and qualitative results indicate that, even though the studies contributed to the present position, the MPH wasn’t an essential requirement for most of the positions in the Health Sector and most of the alumni did not change jobs due to attainment of the MPH degree.
*Theme III* MPH alumni Challenges	1. Finding a job after graduation		*“It’s a really practical profession, it’s not just philosophical studies, it’s really a profession that I have in my hand. And there’s no way to understand where I can fit in work-wise.”* Interview 12 *“I lack knowledge or information as a dietician I know where to look for a job… but in public health, I do not know where to look for a job. Also, today I know less about which jobs I can apply for…”* Interview 5	The qualitative study reveals several challenges in finding a job after the MPH. These challenges can help highlight the quantitative results; most alums do not think the MPH degree helped them find a job due to a lack of information about potential jobs in Public Health. In addition, those who did find a job said the terms of employment and salary were too low and inappropriate for the job. Furthermore, some alumni replied that the MPH was not necessary for changing jobs as many jobs in the Public Health sector do not require an MPH degree.
			*“The person who managed the field before was a nurse who did not have this degree, and the person who replaced me now on maternity leave is also a nurse who does not have this degree.”* Interview 15 *“But we, the health promoters, who graduate from the school of public health, are in dire straits for work. So at least you, as the Ministry of Health, demand it. Demand that it be mandatory…”* Interview 22	
	2. Providing missing competencies potentially could help in promotion in the present position or change of jobs	Do you believe that other competencies, which you did not acquire during your studies, would have been helpful in your present position? Very much 7.1% Moderately 29.1% Little 43.3% Not at all 20.5%	“*You have to understand what the purpose of the degree is if it’s for employment, or if it’s for enrichment or a title. Then it’s different because if it’s for employment, I feel like I do not have enough tools in my hands to process data.”* Interview 18 *“The studies are academic and that’s fine…. But in reality, we must adapt ourselves to the field. Academia is not relevant in the field.”* Interview 22 *“I was missing more management courses. In manpower management, managing strategy determination, in management determining a work plan and its implementation management that is based on employee outputs, employee achievements, and management methods*.” Interview 19 *“Both a bit about a business model and marketing of a private business”* Interview 10	The interviewees highlighted several additional competencies they believe would enhance performance in their present position, including managerial competencies, epidemiological expertise, and marketing competencies. In addition, some of the interviewees mentioned that more practical experience focused on enhancing practical work skills was missing during the MPH studies.
*Theme IV* Information Platform about Public Health Positions.			*“Something that combines useful information and job offers or organizations, which are engaged in health promotion, that I can also access independently”* Interview 3 *“A platform for knowledge sharing, and also for collaborations. And also all kinds of panels that can come out of it”* Interview 6	The public health Internet platform was not covered in the survey but was suggested during the in-depth interviews

### 3.2 Theme I: impact of MPH studies on professional and personal development

Alumni were asked how the MPH program contributed to their personal and professional lives. Around 64% reported that the degree helped contribute to their job and current workplace very much or moderately, and 52% reported that they used the competencies obtained in their studies in their present position very much or moderately. A more in-depth understanding was obtained from the interviews and suggested that the MPH training provided a broad and holistic view of health and specific competencies that graduates could utilize in their workplace. The holistic view included a broader view of health, public health and health systems in Israel and around the world, as well as a broader view of aspects of public health, such as financial, political, organizational, and more. The competencies included managerial and scientific competencies.

### 3.3 Theme II: impact of MPH studies on graduate occupation

The sub-themes included three professional outcomes related to graduation from the MPH program, (a) impact on current job, (b) promotion or increase in salary within the present position, and (c) changing jobs.

The first sub-theme was the application of competencies obtained during studies to the current position. The competencies obtained during the alumni MPH studies provided the ability to approach the graduate’s work more scientifically. The competencies alumni acquired included mainly statistical and scientific tools. In addition, they acquired competencies enabling them to promote the health of the population and competencies to enhance nutritional therapy. The latter was relevant mainly for Health Promotion and Health Nutrition and Behavior sub-specializations graduates. In addition, alumni reported that the studies strengthened their abilities to better address various challenges, such as managing task loads, problem-solving, and overcoming personal and professional challenges. Furthermore, alumni reported that their scientific competencies, such as how to approach research, analytical reading of scientific articles, and seeking reliable data, improved as a result of their studies.

Most respondents did not change their jobs after graduation (80.8%). Therefore the second sub-theme suggests an impact of the studies on promotion or increase in salary at the current job. The quantitative data suggests that 36% received an increase in salary or promotion within their present position. The interviews corroborated this finding, and some said the promotion did not bring with it a salary raise but did increase their job load as additional tasks were given to them.

The third sub-theme included a change of jobs due to MPH studies. Those alumni who did change jobs or positions (19.2%), reported that the change happened due to specific competencies obtained during their MPH studies; these were required for some jobs, such as managerial positions, epidemiology, and health promotion. Exposure to conferences and academic staff helped them find relevant new jobs they were not aware of before; this networking played a major role for those who did find new jobs.

Alumni who did not change jobs or positions reported that many public health jobs did not require an MPH degree even though they were public health positions. Therefore, they were not in a better position to earn these jobs after graduation. This was a recurring complaint of many of the alumni.

### 3.4 Theme III: MPH alumni challenges

The interviewees reported significant challenges in applying the MPH studies and competencies in their careers. They reported challenges in how to advance their careers due to a lack of knowledge and information about where to find a job, what the options were in the public health sector, and where are the available positions. The differences in salary scales between public health and other healthcare professions are seen as not favorable to public health professionals, and therefore, alumni have no incentive to change jobs and move to public health jobs, quite the opposite. This causes disappointment for those with expectations for professional advancement at the end of their studies.

A second major challenge was the missing competencies, with 36.2% reporting on competencies they felt (very much or moderately) were missing from their studies that could have helped them in their current job and in finding a new job. The interviewees highlighted the competencies that were missing including managerial, epidemiological, and marketing competencies as well as practical experience in managing PH challenges.

### 3.5 Theme IV: information platform about public health positions

The study is part of the EU SEEEPHI project wherein one of the goals was to develop a platform to share information about public health positions. Interestingly, some interviewees suggested such a network without prompting, and the responses were recorded. This aligns with alumni suggestions for how best to overcome the hurdle of insufficient information regarding available jobs. All agreed that using Internet social networking platforms could help expose students and graduates to various jobs and positions in the public health arena. In addition, they suggested having public health employment fairs organized by the school or public health organizations. When asked about an internet platform to support alumni job placements, it was very positively accepted, and they stated that it could be very helpful and could be used for additional public health issues.

The public health designed Internet platform was not covered in the survey but was addressed during the interviews. The interviewees shared their perspectives on the required content of the platform and mentioned key topics such as employment, networking, collaboration, public health updates, innovations, conferences, and training opportunities. In addition, they highlighted the potential advantages of this platform, including posting job offers, transparent employment terms, employer profiles, and CV uploads for graduates to facilitate direct job offers. The platform will also enable sharing public health updates, studies, seminars, and conferences. It will showcase successful projects, provide valuable links to public health organizations, and allow organizations to articulate their roles and visions. In addition, the platform could provide access to scientific resources and data, promote information sharing about ongoing or planned studies, and facilitate research collaborations. Additionally, it could foster networking and cooperation between public health professionals, enable consultations across disciplines, and strengthen the connections between universities and graduates.

## 4 Discussion

This mixed methods study looked at MPH alumni in Israel to understand how the graduate degree could help to prepare alumni for and improve their professional career performance. Academic studies aim to broaden knowledge and skills and increase cognitive and practical abilities that can then be applied in the graduates’ professional lives, generally (20) and specifically in public health (21).

Our findings suggest that the MPH degree studies provide a holistic public health perspective that students did not acquire in their undergraduate studies. It should be noted that undergraduate studies for the vast majority of students embarking on an MPH program are directed at acquiring a healthcare profession, such as nursing, medicine, or nutrition, and they have limited exposure to public health in their undergraduate studies. Other studies also suggest that academic studies of public health enhance the student’s roles in areas such as public health policy analysis, planning, implementation and evaluation, leadership, and research (22) and acquiring specific competencies for public health (7).

However, many of the respondents did not feel their MPH studies helped them improve their job position or income, which led to disappointment. A minority of respondents reported major changes in their employment. The study suggests three major outcomes, including improvement in their job performance, change in employment for the better and increases in salary or position. Two-thirds of alumni reported improvements in their current job performance, however, less than 20% reported changing jobs as a result of their studies. Over 60% reported little or no increases in salary or position. It must be noted that the MPH studies were intended to help students improve their ability to use the skills that were taught and not intended to help increase their salary or change jobs, even though this often was the expectation of many students.

In a review of the subject, Krasna et al. found that few studies focused on employment outcomes as we did in this study (21). Buunaaisie et al. (22) did look at employment in an international program in England and found that 63% of graduates were employed 1 year after graduation. However, our student characteristic is different as most of them are employed before joining the program, but only a third report improving their employment.

A few barriers that may prevent improving alumni in the workforce were identified. A major barrier is that salaries in the public health workforce are low compared to salaries of workers in healthcare roles. The public health domain does not have a strong union, successful in demanding increases in salaries, and therefore transferring to a public health position frequently does not come with a salary increase, even if it is a better position. We did not find this barrier reported in other studies, this may be that in other countries public health jobs are paid well or that the other studies interviewed graduates and not alumni.

As an additional barrier, some positions in public health did not require the MPH degree as a prerequisite, so there is little incentive to transfer to a public health position especially for low level positions. In a study in Germany, Arnold et al. (23) reported that they had considerable difficulties in attracting well-qualified personnel to the public health services and identified barriers to increasing the attractiveness of public health as a career.

Our study demonstrated that less than half the alumni saw improvements in their position or salary after graduation, however, we need to consider the large range of follow-up time since graduation in our sample. These results suggest our school needs to provide students with better information regarding positions available for MPH graduates, as the interviewees suggested in the in-depth interviews (24, 25).

Previous studies looked at MPH alumni’s opinions regarding their studies but less so regarding their work performance as a result of their studies. Le et al. (26) interviewed 187 graduates in Hanoi, which reported high rates of developing relevant skills and satisfaction, however, they felt the MPH emphasized research methods at the expense of management and operational competencies.

There is a great need to better understand how best to enhance the compatibility between public health training program competencies and the implementation of competencies required by employers to address emerging public health needs in Israel (9). Therefore Baskin et al. interviewed 49 PH managers in an attempt to understand this. The authors found deficiencies in all categories of PH competencies. Employers reported they need better-trained graduates and that the PH graduates were deficient in essential competencies, concluding that there is an urgent need for PHW to have the ability to deal with increasingly complex and diversified jobs (15). In this study alumni reported missing competencies, such as managerial, epidemiological, and marketing competencies. These gaps between curricula and work needs could be due to the changing public health landscape and newer developments in technology and the workforce requiring a combination of multiple skills from several disciplines.

Other studies also looked at deficiencies in the MPH programs, in Canadian MPH programs, there also seems to be a lack of courses addressing core competencies, especially diversity and inclusiveness communication and leadership (27). As a result of social and economic changes, the Boston University public health department redesigned the program (28). They aimed to “provide students with integrated foundational knowledge; specialized skills and training in key areas sought” with the aim to close gaps between competencies learned in the program and those needed by employees.

Closing these mentioned gaps in competencies of graduates in a changing and complex public health landscape may help promote finding jobs and improve job position or salary post MPH graduation. This may strengthen the argument of the importance and necessity of MPH graduates in the health system.

There is room for revamping the public health curricula in Israel, both adding competencies alumni reported missing and building an accessible platform with information regarding available PH jobs.

### 4.1 Limitations

This study is exploratory and has several limitations. First, it was performed in only one school of public health in Israel, and therefore may not represent the total alumni of public health schools in Israel, even though we are not aware of any differences between the schools except for their geographical location. This study can serve as a case study focusing on a single school, similar to many other studies on this subject, as pointed out in a scoping review (21). It can help build a more complete picture of gaps and opportunities in public health education. As part of the study is qualitative the importance of the representativeness is less of a limitation as the information collected gives an in-depth view of the subject.

Second, the sample size was not large and the response rate was low, therefore the level of representativeness is not clear. However, we did approach the total population of learners relevant to the study’s goals and made efforts to get a reasonably representative sample of our alumni at least by gender, age and time from graduation. As this is a mixed methods approach, the addition of the qualitative interviews provides an in-depth view of the outcomes and validates the quantitative results, somewhat overcoming the partial representativeness of the sample by extracting themes from the alumni. Explanations for the low response rate may be that there is no ongoing email follow-up with alumni due to restrictions imposed by the Israeli Communications Law (commonly known as the “Spam Law”), which sets strict regulations on sending electronic communications. That said, response rates for email surveys targeting healthcare professional alumni, tend to be low (29), perhaps influenced by the increasing volume of email communications healthcare professionals receive, their busy schedules, and the lack of personal incentives.

Third, the study results reflect the Israeli health and higher education systems, which may not be generalized to other countries with their distinct public health delivery and training systems, comprising unique organizational characteristics, barriers to job hires, and different clinical and political settings. It is unclear to what degree this study can be generalized to other countries even though the curriculum of the Haifa University Public Health School adheres to the WHO-ASPHER Competency Framework (6). However, the Israeli academic system does not differ significantly from systems in Europe and America, therefore we think the results can contribute to understanding the place of the MPH studies for the workforce and the PH services.

Fourth, to address potential validity threats, we made all efforts to ensure methodological rigor by using a standardized codebook, meeting frequently, sharing and comparing our results, and performing a pilot analysis.

Fifth, the quotes chosen for Table 2 were translated from Hebrew to English and back-translated to ensure their correct meaning.

Finally, we did not measure objective occupational measures like salaries of graduates versus undergraduates or the impacts that alumni had on society, which can be tested in future studies.

Further studies are needed to identify specific groups with specific barriers and problems and follow up these alumni and see how their career playouts in the long run. In addition, there is also a need to compare and correlate the results with opinions of the employers that employ the graduates to verify the significance of the opinions expressed in this study. This would serve as a validation of the study. This calls for further studies not only among alumni but also among employees and employers in the public health services and curative healthcare services. Also, looking at how the public health services changed over time regarding the needs required from graduates could teach us of trends in the public health workforce.

## 5 Conclusion

Public health graduate studies at the University of Haifa School of Public Health in Israel are successful in providing a holistic view of health and the healthcare system and basic competencies for the workforce. However, alumni reported less satisfaction in enhancing job opportunities. There is a need to align the curricula with the needs of the PHW and increase the ability of alumni to utilize their acquired skills and competencies more effectively. The study also suggests there is a need to develop pathways between the academy and field to translate graduate studies into occupational promotion and economic revenue. Future studies are needed to evaluate and enhance MPH degree learning outcomes and create a better-prepared and resilient workforce.

## Data Availability

The raw data supporting the conclusions of this article will be made available by the authors, without undue reservation.
