# Supplementary material for: Professional development: a mixed methods study of Masters of Public Health alumni
Source: Front Public Health. 2024 Oct 23;12:1429474. doi: 10.3389/fpubh.2024.1429474 (PMC11537938; doi:10.3389/fpubh.2024.1429474)
Supplement: Supplementary file 1 [file Table_1.DOCX]

**Study questionnaire**

1. **Gender:** Male / female/ other
2. **What is your age?** ______________
3. **What is your first degree in?** ___________________
4. **What is the last academic degree you completed at our school? (You can indicate more than one)**

- Master's degree (MPH, MHA, MAN)
- Doctorate (Ph.D.) (Do not indicate if you are an active student at this time)

1. **Study major:** (Please select)

- Health nutrition and behavior (1)
- Health systems management (2)
- Public health
- Epidemiology (3)
- Biostatistics (4)
- Director of Health Systems (5)
- Health Promotion (6)
- Community health (7)
- Environmental health and occupational health (8)
- Mental health services (9)

1. **In what year did you graduate from the School of Public Health?** ______________
2. **Since graduating from our institution, have you continued your higher education at another institution?** Yes / No (if not, go to question 8)
   1. **In which institution?**

- Tel Aviv University
- Bar Ilan University
- Technion
- Weizmann Institute of Science
- Ben Gurion University
- The Hebrew University
- Ariel University
- College or Open University
- A university abroad
  1. **Which degree?**
- Master's degree (yes/no)
- Doctorate (Ph.D.) (yea/no)
  1. **In which field?**
- Public health
- Medicine
- Paramedical
- Research methods
- Social Sciences
- Other, please specify: ___________

1. **What platform did you use to find your current job? (You can indicate more than one)**

- Job board
- Self-application to the workplace
- Through a call or ad posted by the workplace
- Social media
- With the help of a friend
- With the help of college
- Recruitment Conference
- Other: _________________

1. **Where are you currently working? (You can mark more than one answer)**

- Health service )HMO(
- Hospital
- Pharmacy
- District Health Bureau
- Ministry of Health
- Family and baby health center ([Tipat Chalav](https://context.reverso.net/%D7%AA%D7%A8%D7%92%D7%95%D7%9D/%D7%90%D7%A0%D7%92%D7%9C%D7%99%D7%AA-%D7%A2%D7%91%D7%A8%D7%99%D7%AA/Tipat+Chalav))
- Pharmaceutical company
- Ph.D. student
- Research Institute
- Private clinic
- A high-tech company or start-up
- Other, please specify: ___________________

1. **What is your role in your workplace?** ___________________
2. **How many years have you worked at this place?** ___________________
3. **Have you changed jobs after receiving your degree**? Yes/ No
4. **Have you changed your job definition after receiving your degree**? Yes/ No
5. **There are six multiple-choice questions in the following questionnaire regarding the benefits of your studies about your employment. Please mark the most relevant answer for you.**

|  | Not at all (1) | Little (2) | Moderately (3) | Very much (4) |
| --- | --- | --- | --- | --- |
| 14.1 To what extent did the degree you studied help you find your current job? |  |  |  |  |
| 14.2 How much do you feel you work in a workplace-related to the degree you studied? |  |  |  |  |
| 14.3 To what extent does the degree you studied help you to contribute to your current workplace? |  |  |  |  |
| 14.4 To what extent have the skills you obtained during your studies (such as statistical analysis, scientific writing, critical science reading) contributed to you in your current workplace? |  |  |  |  |
| 14.5 How much do you think skills you did not acquire during your studies would be helpful in your current job?  (If there are any - we would be happy if you could list them in question 15 below) |  |  |  |  |
| 14.6 To what extent did the degree you studied help you get a promotion (in rank or salary) in your current job? |  |  |  |  |

1. **Any comments or additional data you want to add:** __________________________
2. **If you agree that we will contact you for an in-depth interview (approximately 30 minutes) about your studies and your current work, please enter the link, fill in your details, and we will contact you later. The identifying information that will be filled in at the link cannot be associated with your answers to this questionnaire.**

**Thank you very much for filling out the questionnaire!**

**Qualitative questionnaire for in-depth interviews**

Part I - General details

1. What is your age?
2. What academic degree did you enroll for at the School of Public Health?
3. What was your sub-specialization of study in the master's degree?
4. What year did you graduate from the School of Public Health?
5. Where is your current position?
6. What is your role at work?
7. How did you get this job?
8. Have you changed your job since/after graduation? Or did you change your job position?

Part II in-depth questions

1. How did the master’s degree help you find your current job or help you get your current position?
2. What competencies did you acquire during your degree that contributed to your present job?
3. What were your considerations when choosing your current workplace?
4. What competencies did you hope to acquire during your studies but didn't?
5. What other things would you add/change in the program to make it more effective for entering the public health workforce?
6. What things in general (not necessarily related to the MPH) could help you find a job relevant to your field of study?
7. Suppose we set up a platform that would support the public health community, including a feature that would allow contact between employers and employees. What should be there?
8. How could the School of Public Health alumnae community of contribute to you personally and professionally?
9. Do you have any clarifications or comments to add?
