# Supplementary material for: Professional development: a mixed methods study of Masters of Public Health alumni
Source: Front Public Health. 2024 Oct 23;12:1429474. doi: 10.3389/fpubh.2024.1429474 (PMC11537938; doi:10.3389/fpubh.2024.1429474)
Supplement: Supplementary file 2 [file Data_Sheet_1.PDF]

Translation from Hebrew

Date:______________

Explanatory page for study participants

Dear alumni,
We are asking you to participate in a study, conducted by the School of Public Health at the University of Haifa, among school graduates. The study is led by Prof. Shira Zilber-Sagi and Prof. Orna Baron-Epel, as part of a project to promote the public health workforce. The study is in collaboration with the Erasmus Program and the European Association of Schools of Public Health.

1. **Purpose of the study:**

The goal of the study is to map the needs of graduates of the School of Public Health at the University of Haifa when entering the labor market and finding work.
The study will serve as the basis for building an international online platform for connecting students and alumni, public health professionals and workplaces. The purpose of the platform is to provide information about public health jobs, to make job offers accessible to students and graduates, and to enable officials in public health institutions to recruit employees. Your answers to the following questions will help us understand your needs.

2. **What does the study include?**

The study includes a one-time participation in an in-depth interview on Zoom or face-to-face, according to your preference. The interview will last about 30 minutes, will be recorded by audio recording (via Zoom or with the help of a voice recorder) and will be saved until transcribed. After the transcription, the recording will be deleted. Your consent to participate in the study will be recorded at the beginning of the interview and will remain documented as part of its transcription.

3. **The benefits to you and or others expect as a result of the study:**

You are not expected to have benefits as a result of participating in the study.

4. **Are there any risks/discomfort that may accompany participation in the study?**

No risks or discomfort are expected as a result of participating in the study.

5. **Can my participation in the study be terminated at the initiative of the researcher?**

No

6. **Do I have to participate?**

   It is important for us to clarify that participation in the study is voluntary and you do not have to participate in the study. Your refusal to participate will have no consequences for you and will not harm you in any way.

7. **Can I stop participating in research in the middle?**

   You can terminate your participation in the study at any time, without any consequences for you. You can skip questions you don't want to answer.

8. **Reward:**

There is no reward for participating in the study.

Confidentiality and data retention: Recordings will be kept securely until they are transcribed. After the transcription, the interview recording will be deleted. The transcription will be preserved in a coded manner. Your identifying information will be stored securely, will be disclosed only to research staff members, and will not be published in any scientific or other publication.

Inquiries

Dr. Dana Ivankovsky Wachman Phone for inquiries: 050-8614759

Email Address: divancov@campus.haifa.ac.il

Sincerely,

Prof. Shira Zilber-Sagi

Prof. Orna Baron-Epel

and the research team

e
